# Occurrence of ovarian follicular dominance during stimulation for IVM impacts usable blastocyst yield

**DOI:** 10.5935/1518-0557.20180006

**Published:** 2018

**Authors:** Sergio Romero, Ricardo Pella, Francisco Escudero, Ygor Pérez, Mario García, Patricia Orihuela

**Affiliations:** 1 Centro de Fertilidad y Reproducción Asistida CEFRA, Dirección de Investigación, Lima, Peru.; 2 Centro de Fertilidad y Reproducción Asistida CEFRA, Laboratorio de Embriología, Lima, Peru.; 3 Centro de Fertilidad y Reproducción Asistida CEFRA, Dirección Médica, Lima, Peru.

**Keywords:** PCO, IVM, follicular dominance, blastocysts, pregnancy

## Abstract

**Objective:**

To evaluate the influence of ovarian follicular dominance on the outcome of oocyte *in-vitro* maturation.

**Methods:**

This retrospective cohort study included 21 patients with polycystic ovaries or polycystic ovary syndrome (Rotterdam criteria, 2004) subjected to 24 *in-vitro* maturation (IVM) cycles between October 2015 and January 2017. Patients undergoing IVM received minimal gonadotropin stimulation starting on day 2 or 3 of the cycle; ovum pick-up typically occurred on days 6 to 8. No hCG-trigger shot was given. Following 30h of IVM, mature oocytes were inseminated by ICSI and the resulting embryos cultured up to the blastocyst stage.

**Results:**

Ovarian follicular dominance was observed in nine of the 24 IVM cycles. Oocyte IVM yielded an overall maturation rate of 69.3±23.8%, and no difference was observed when the groups with or without a dominant follicle were assessed independently. The rates of fertilization and usable blastocysts per fertilized oocyte, mature oocyte (Metaphase II) or cumulus-oocyte-complex were nearly three times higher (28.7±22.5%) in the group without ovarian follicular dominance. No differences were found in the clinical pregnancy rates attained by the individuals with or without a dominant follicle after 21 vitrified-warmed blastocyst transfer cycles.

**Conclusion:**

Occurrence of ovarian follicular dominance during hormonal stimulation for *in-vitro* maturation negatively impacted embryological outcomes. Strategies devised to limit the appearance of ovarian follicular dominance must be further explored.

## INTRODUCTION

Oocyte *in-vitro* maturation (IVM) is a poorly disseminated assisted reproductive technology, especially because it has been perceived as a procedure with suboptimal results in comparison to traditional ICSI. Although IVM offers multiple benefits (in particular, for the patient), many embryologists and clinicians are still reluctant to consider it as an alternative ART procedure. This limits the amount of clinically relevant data and impairs the development of evidence-based enhancements to the procedure.

The use of IVM is mostly indicated for patients with polycystic ovaries (PCO) or polycystic ovary syndrome (PCOS). When undergoing ovarian stimulation with gonadotropins (in fertility treatment), patients suffering from either of the conditions are at risk of developing ovarian hyperstimulation syndrome (OHSS), a potentially dangerous condition (MacDougall *et al.*, 1993; Brinsden *et al.*, 1995). While risk is diminished, albeit not eliminated, when patients are administered a GnRH agonist trigger (Santos-Ribeiro *et al.*, 2015), the principle of IVM poses nearly no risk of inducing OHSS. Furthermore, due to its nature, IVM is a valuable tool for preserving fertility in cancer patients in which hormonal stimulation must be avoided or limited (De Vos, 2016; Wang *et al.*, 2016).

Most IVM protocols make use of mild ovarian stimulation to induce follicular growth. However, prior to oocyte retrieval they may differ on whether hCG is used as a trigger or not. The hCG trigger shot initiates the process of maturation within the follicle (in vivo), producing three populations of oocytes at the time of retrieval (Metaphase II, Metaphase I, and germinal vesicle oocytes). While MII oocytes can undergo ICSI immediately, metaphase I and GV oocytes require *in-vitro* maturation, which requires the addition of 2 or 3 time points for ICSI(Son *et al.*, 2011). Non-hCG-triggered protocols regularly produce 100% immature GV oocytes, and ultimately constitute the most adequate source for IVM.

Few scientific reports have been published in South America on the use of IVM, either on rescuing immature oocytes obtained from stimulated cycles for *in vitro* fertilization (Braga *et al.*, 2010; Vieira *et al.*, 2011; Alcoba *et al.*, 2015) or on the use of IVM with intention-to-treat patients (Bos-Mikich *et al.*, 2011; Lucena & Moreno-Ortiz, 2013; Tominaga *et al.*, 2015). Only one study described a non-hCG-triggered protocol followed by transfer of vitrified/thawed cleavage stage embryos (Tominaga *et al.*, 2015).

To the best of our knowledge, this is the first scientific report from the region to look into the use of IVM as an alternative ART procedure with the intention to treat patients suffering from PCO/PCOS and the transfer of vitrified/thawed blastocysts in deferred cycles. Our preliminary findings provide insight into the negative effects of ovarian follicular dominance in the preparations for IVM.

## MATERIALS AND METHOD

### Patient selection

*In-vitro* maturation is the most recently implemented assisted reproductive technology (ART) procedure in our IVF center. This retrospective study included 21 patients with polycystic ovaries (PCO) or polycystic ovary syndrome (PCOS) according to the Rotterdam criteria (Rotterdam ESHRE/ASRM-Sponsored PCOS consensus workshop group) subjected to 24 *in-vitro* maturation (IVM) cycles between October 2015 and January 2017.

The patients had baseline ovary scans performed between days 1 and 3 of their cycles to rule out the presence of cysts. A new evaluation was repeated between days 5 and 8 to image and measure follicle size.

Routine hormonal analysis for IVM has not been standardized yet at our center, therefore whenever considered appropriate by the treating specialist, tests for AMH, FSH, LH, and E2 were ordered and the outcomes recorded. Most of the tests were ordered on days 1 to 3 (baseline) and days 5 to 8 of the cycle, when the decision for ovum pick-up (OPU) was made.

### Oocyte retrieval, fertilization, embryo culture and transfer

Patients undergoing IVM received minimal gonadotropin stimulation (3 to 4 doses of hMG, the equivalent to 225 IU daily) starting on day 2 or 3 of the cycle. When most follicles reached 9-10mm, ovum pick-up was scheduled for at the latest 45 hours after the last gonadotropin injection (usually on days 6 to 8). No hCG trigger shot was given.

Ovum pick-up was performed with a 16-gauge aspiration needle at an aspiration pressure of 80mmHg. Cumulus-oocyte-complexes (COCs) were placed in a 4-well dish (Oosafe^®^) containing 750µl of Global^®^ medium for fertilization (Life Global) supplemented with 75mIU/ml of FSH (Gonal-F^®^, Merck-Serono), 100mIU/mL of hCG (Choragon^®^, Ferring Pharmaceuticals; or Pregnyl^®^, Organon), and 10% patient heat-inactivated serum, and were cultured at 37°C in 6% CO_2_ for 30h. After maturation, the oocytes were denuded of cumulus cells following a brief exposure to hyaluronidase and by repeated pipetting.

Oocytes were subsequently inseminated by ICSI with spermatozoa from patient partners and placed in Global^®^ Total^®^ at 6% CO_2_ and 5%O_2_ in air, at 37°C. Fertilization was assessed 16 to 18hrs after ICSI and presumptive embryos were further cultured up to the blastocyst stage. On days 5 or 6, after embryological assessment, top and good quality embryos (usable embryos) were vitrified for transfer in a deferred natural or artificial cycle.

### Statistics

Percentages of maturation, fertilization, usable blastocysts per fertilized oocyte, mature oocyte, and COC were calculated for each cycle. Statistical comparisons of such proportions for the two groups (with or without dominant follicle) were performed after arcsine conversion. Significant differences were calculated with the Mann-Whitney test.

Significant differences for pregnancy outcome (number of transferred embryos, biochemical pregnancy, clinical pregnancy etc.) were elicited using Fisher's exact test.

Statistical analyses were performed using GraphPad Prism version 7.00 for Mac OS X, GraphPad Software, La Jolla California USA, www.graphpad.com.

## RESULTS

None of the patients included in this study had previously undergone IVM. Twenty-one patients underwent a total of 24 IVM cycles. Table 1 describes patient characteristics.

**Table 1:** Characteristics of patients included in the study

Number of cycles	24
Age (mean±SD)	32±2.7
BMI (mean±SD)	25.6±3.0
AMH (n=16) (ng/mL, mean±SD)	6.8±4
Duration of stimulation (days, mean±SD) [range]	3.6±0.5 [3-4]
Gonadotropin dose (IU, mean±SD) [range]	828.3±118.5 [675–1050]

The patients were asked to contact the center within the first two days of their cycles to schedule a baseline ovary scans and blood tests. Hormonal baseline parameters are shown in Table 2. Following mild and short hormonal stimulation, the mean peak estradiol level was 563.1±306.3 (mean ± SD).

**Table 2:** Patient baseline hormonal parameters on days 1 to 3 of the cycle

FSH (n=14) (mIU/mL, mean±SD)	6.1±1.2
LH (n=15) (mIU/mL, mean±SD)	11.6±11.5
Estradiol (n=15) (pg/mL, mean±SD)	37.1±13.4

While the negative effects of dominant follicles on traditional ART procedures have been suggested, in IVM the concept is still poorly defined. In order to investigate this matter in our small population, growing follicles were arbitrarily deemed dominant when they were at least 2 mm larger than the mean size of the rest of the cohort at the time of oocyte retrieval. Dominant follicles were observed in nine of the 24 IVM cycles; ovarian follicular dominance was not seen in 15 cycles.

As shown in Table 3, the overall maturation rate was 69.3±23.8%; this proportion was not affected when maturation was assessed independently for COCs obtained from ovaries either with or without a dominant follicle. However, fertilization was significantly higher in the group without a dominant follicle.

**Table 3:** Embryology outcomes

Embryology outcomes	Overall	(+) Dominant follicle	(-) Dominant follicle	*p*-value*
Number of oocytes [range per patient]	239 [4–42]	115 [4–42]	124 [4–15]	-
Number of mature oocytes after IVM	164	80	84	-
Percentage of mature oocytes, per cycle (mean±SD)	69.3±23.8%	69.7±16.2%	69.0±27.9%	0.8257
Percentage of fertilized oocytes, per cycle (mean±SD)	88.8±15.2%	82±14.3%	92.9±14.6%	0.0454
Percentage of usable blastocyst/fertilized oocyte (mean±SD)	37.7±28.3%	20.5±19.1%	48.0±28.3%	0.0161
Percentage of usable blastocyst/MII oocyte (mean±SD)	34.3±28.5%	16.4±15%	45.0±29.6%	0.0076
Percentage of usable blastocyst/COC (mean±SD)	21.9±20.6%	10.6±10.6%	28.7±22.5%	0.0217

* Statistical calculations were performed to compare the outcomes of patients in which a dominant follicle was present (+) or absent (-)

Similarly, the rates of usable blastocysts per fertilized oocyte, mature oocyte (MII) or COC were significantly higher in the group without dominant follicles.

To avoid any potential asynchrony between embryo development and endometrial receptivity (which may occur if a fresh transfer were performed), all embryo transfers were carried out in deferred cycles. Twenty-one transfers of vitrified-warmed blastocysts were performed. Eight of them were carried out with embryos from the (+) dominant follicle group, while 13 had embryos from the (-) dominant follicle group.

Table 4 shows the data for pregnancy outcomes and elicits that no differences were observed between the two groups.

**Table 4:** Pregnancy outcome

Frozen embryo transfers	Overall		(+) Dominant follicle		(-) Dominant follicle		*p*-value^†^
	n=21	%	n=8	%	n=13	*%*	*-*
Nº of embryos transferred	1.57±0.51	-	1.5±0.5	-	1.6±0.5	-	0.673
Biochemical pregnancies	17/21	81.0	7/8	87.5	10/13	76.9	>0.99
Clinical pregnancies	13/21	61.9	5/8	62.5	8/13	61.5	>0.99
Live birth^‡^	7/13	53.8	4/5	80	3/8	37.5	-
Ongoing pregnancy^‡^	4/13	30.8	1/5	20	3/8	37.5	-
Miscarriage^‡^	1/13	7.7	0/5	0	1/8	12.5	-

† Statistical calculations were performed to compare the outcomes of patients in which a dominant follicle was present (+) or absent (-)

‡ Rates of live birth, ongoing pregnancy and miscarriage are only shown as reference.

## DISCUSSION

Ovarian follicular dominance is a naturally occurring event during the menstrual cycle (Pache *et al.*, 1990), however during controlled ovarian stimulation, efforts are directed towards its avoidance, as it has been suggested to be a negative factor for the outcome of ART procedures (Yoldemir *et al.*, 2011).

During IVM procedures, it is a common practice to perform OPU before the follicles become larger than 12-13mm, thus limiting the potential negative effects on the rest of the cohort. Most centers prefer to schedule OPUs when the largest follicle reaches 10mm (Mikkelsen *et al.*, 1999; Russell, 1999; Le Du *et al.*, 2005; Söderström-Anttila *et al.*, 2005; De Vos *et al.*, 2011). However, the only evidence that a dominant follicle (larger than 14mm) affects IVM outcomes comes from a study in hCG-triggered IVM cycles (Son *et al.*, 2008a). There currently is no evidence to state that the presence of a dominant follicle in a non-hCG-triggered IVM cycle might affect the rest of the cohort. Therefore, our study produced the first data on the matter for non-hCG-triggered IVM cycles.

Implantation rates following IVM were reported to be below 15%, as reviewed by Smitz et al. (2011), particularly when embryos are transferred at the cleavage stage in fresh cycles (Son *et al.*, 2008b; De Vos *et al.*, 2011). Moreover, since endometrial preparation for fresh embryo transfer following IVM proved to be challenging, deferring embryo transfer to a subsequent artificial cycle has been suggested as an appealing strategy (Ortega-Hrepich *et al.*, 2013).

In the study presented here, all patients had been diagnosed with PCO or PCOS; consequently, all were stimulated for follicular growth with brief low-dose gonadotropin therapy. This stimulation protocol was enough to drive follicular growth and to induce modest increases in the production of estradiol, whose levels are lower than what has been described in other conventional ART stimulation protocols (Smitz *et al.*, 2007).

The non-hCG-triggered stimulation and the IVM protocols used in this study yielded a good maturation rate (~70%), consistent with what was described by others using similar approaches (Junk & Yeap, 2012; Walls *et al.*, 2015). Similarly, the overall rates of usable blastocyst per fertilized oocyte (38%), per mature oocyte (34%) or per COC (22%) were quite comparable to the rates described by Walls *et al*. (2015) and Junk & Yeap (2012).

Nevertheless, the negative impacts of dominant follicles on the rest of the cohort following a non-hCG-triggered stimulation were described here for the first time. In fact, the yield of usable blastocysts per COC increased by almost three times (11% *vs.* 29%) when a dominant follicle was absent. Since the maturation rate was not affected by the presence/absence of a dominant follicle, it is tempting to suggest that the presence of a dominant follicle does not affect meiotic capacity (meiotic competence), while it impairs the quality of the oocytes (developmental competence) of a follicular cohort.

In summary, the assessed embryological parameters revealed that oocytes unexposed to the influence of dominant follicles yielded larger numbers of blastocysts than oocytes affected by ovarian follicular dominance. However, preliminary data on clinical pregnancy rates indicated that once blastocysts are formed, their potential to produce pregnancy is not affected by the origin of the oocytes.

## CONCLUSION

The data presented herein suggest that ovarian follicular dominance during hormonal stimulation for IVM negatively impacts embryological outcomes. Therefore, strategies devised to limit the appearance of ovarian follicular dominance must be further explored.
